# Climate change will reduce suitable Caatinga dry forest habitat for endemic plants with disproportionate impacts on specialized reproductive strategies

**DOI:** 10.1371/journal.pone.0217028

**Published:** 2019-05-29

**Authors:** Jéssica Luiza Souza e Silva, Oswaldo Cruz-Neto, Carlos A. Peres, Marcelo Tabarelli, Ariadna Valentina Lopes

**Affiliations:** 1 Programa de Pós-Graduação em Biologia Vegetal, Departamento de Botânica, Universidade Federal de Pernambuco, Recife, Pernambuco, Brazil; 2 Departamento de Botânica, Universidade Federal de Pernambuco, Recife, Pernambuco, Brazil; 3 School of Environmental Sciences, University of East Anglia, Norwich, United Kingdom; UNAM, MEXICO

## Abstract

Global climate change alters the dynamic of natural ecosystems and directly affects species distributions, persistence and diversity. The impacts of climate change may lead to dramatic changes in biotic interactions, such as pollination and seed dispersal. Life history traits are extremely important to consider the vulnerability of a species to climate change, producing more robust models than those based primarily on species distributions. Here, we hypothesized that rising temperatures and aridity will reduce suitable habitats for the endemic flora of the Caatinga, the most diverse dry tropical forest on Earth. Specifically, species with specialized reproductive traits (e.g. vertebrate pollination, biotic dispersal, obligatory cross-pollination) should be more affected by climate change than those with generalist traits. We performed two ecological niche models (current and future) to simulate the effects of climate change on the distribution area of endemic species in relation to life-history traits. We used the MIROC-ESM and CCSM4 models for both intermediate (RCP4.5) and highest predicted (RCP8.5) GHG emission scenarios, with a resolution of 30' (~1 km^2^). Habitat with high occurrence probability (>80%) of endemic species will be reduced (up to ~10% for trees, ~13% for non-arboreous, 10–28% for species with any pollination/reproductive system), with the greatest reductions for species with specialized reproductive traits. In addition, the likely concentration of endemic plants in the extreme northeastern portion of the Caatinga, in more mesic areas, coincides with the currently most human-modified areas of the ecosystem, which combined with climate change will further contract suitable habitats of endemic species. In conclusion, plant species endemic to the Caatinga are highly vulnerable to even conservative scenarios of future climate change and may lose much of their climatic envelopes. New protected areas should be located in the northeastern portion of the Caatinga, which hosts a more favorable climate, but is currently exposed to escalating agricultural intensification.

## Introduction

At a global scale, climate change is mainly represented by increases in temperature and divergent changes in precipitation regimes, which will likely become more variable and extreme [1, 2]. Several tropical biomes are predicted to experience extreme climatic changes, which may result mainly in increased aridity [3, 4]. The degree to which an ecosystem changes and its ability to recover to original conditions depends on the intensity of exposure, with different terrestrial regions exhibiting varying vulnerability to climate change [5]. In South America, novel climate-vegetation equilibrium conditions are predicted, in which savannas may replace some tropical rainforest areas and semi-desert areas may replace much of the drought polygon of northeastern Brazil (geographic polygon bound by the annual 800-mm isohyet) [6]. In response to these emergent climatic conditions, species may persist within their original distribution range due to acclimatization and phenotypic plasticity, migrate to new areas of suitable climatic conditions, or may undergo local extinctions (e.g. [7, 8]). Indeed, changes in climate regimes may act as an important historical driver of natural selection [9]. By affecting organisms from single populations to entire biomes, climate change may exhibit a broad footprint in divergent levels of biological organization across different regions on Earth [5, 10].

One of the fastest responses observed for distinct groups of organisms is a shift in their distributions to track the emergent distribution of suitable habitat conditions (e.g. [11]). In this context, species may undergo shifts in their latitudinal and altitudinal ranges resulting in expansion, contraction or fragmentation of their original distributions [10]. These distributional changes may be also followed by (1) changes in ecological interactions, such as pollination [12–14], seed dispersal [15], and herbivory [16, 17], (2) spatial and temporal mismatches in soil nutrient dynamics [18, 19], (3) changes in niche space [20], (4) species invasions [21–24] and (5) evolutionary changes, such as adaptation or local extinction [25, 26]. In addition, other changes in morphology, such as reductions in body mass and alterations in shape, colour and brightness (e.g. [10]), and reproductive phenology, such as earlier flowering in plants (e.g. [27]), are expected in many climate change scenarios. Therefore, by interfering with ecological interactions, climate change can detrimentally affect global biodiversity and the flow of ecosystem functions and services [28].

Life history traits are closely related to species performance in communities and ecosystems [29–32]. In this sense, they can be used as predictors of species responses to environmental changes, including changes in land use (e.g. habitat loss and fragmentation) [33, 34], livestock grazing [35], fire [36]), and climate [37, 38]. These traits are extremely important to consider the differential vulnerability of species to climate change since they ensure more robust (trait-based) models than studies based primarily on species distribution (correlative models) (see [37, 39] for a review). The combination of trait-based models with correlative models may offer a good opportunity for a better understanding of species vulnerability to climate change (see [39] and references therein for a review). Traits such as reduced dispersal capacity, slow reproductive rate (trees), pollination and dispersal by bats, specialized diet or habitat, narrow physiological tolerance range, low adaptive potential, restricted distribution, and population rarity, among others, can all render a species more vulnerable to climate change (e.g. [37–40]). In general, species with specialist life history traits (i.e. traits with some degree of ecological specificity/restriction *sensu* [41]), such as those cited above, play a key role in ecosystem processes [42]. Many studies have shown that these traits/strategies are mainly observed in endemic species or species showing some level of vulnerability (e.g. [43–49]). The vast majority of these studies (~70%) were developed in North America, Europe or Australia. Neotropical regions, which hold most of the global biodiversity, including endemic species, have been therefore neglected [40]. Angiosperms, birds and mammals are the most studied taxonomic groups [40] and forecasts suggest severe species losses in these taxa globally [43], particularly in biodiversity hotspots [44], and mainly specialist species [50].

The Caatinga Phytogeographic Domain (CPD) is a seasonally dry tropical forest (SDTF) endemic to Brazil and represents one of the largest semiarid regions in South America, occurring over approximately 800,000 km^2^ [51]. The Caatinga is located in northeastern Brazil, abutting the Atlantic forest domain to the east and the Cerrado to the west and south. The seasonality and rainfall distribution, associated with elevated temperatures and highly variable edaphic conditions, drive a diverse spectrum of Caatinga phytogeographic formations (e.g. [52–55]). These range from open areas with shrub and herbaceous vegetation, such as the inselbergs, to areas where tree species predominate in both species’ richness and abundance, such as arboreal Caatingas (*sensu* [56]). The Caatinga flora is considered the most diverse SDTF on Earth, harboring 298 endemic species [57], representing 31 genera of flowering plants [58]. Future climatic conditions for the Caatinga vegetation cover indicate that some regions will likely experience high levels of aridity and subsequent desertification [4, 59], resulting in changes in plant species diversity and distribution [1, 6] and key ecological processes such as pollination and associated functions [13]. Due to the characteristics mentioned above, the Caatinga represents an ideal tropical model for studies on the effects of climate change on species distributions.

Here, we seek to understand the effects of climate change on the range and distribution of suitable habitats for flowering plants endemic to the Caatinga, in relation to growth habit (arboreous and non-arboreous) and reproductive traits (e.g. pollination, reproductive systems, dispersal mode). Thus, we used what Foden *et al*. [39] very recently outlined as a trait-correlative approach for assessing the vulnerability of species to climate change. First, we test the hypothesis that areas with highly suitable climatic conditions for endemic flowering plants will be reduced under scenarios of climate change represented by increases in temperature and decreases in precipitation. We also test the hypothesis that elevated temperatures and reduced precipitation will promote greater reduction in the suitable habitat for endemic plants with arboreous habits and specialized reproductive strategies (e.g. vertebrate pollination, biotic dispersal, obligatory cross-pollination) compared to plants exhibiting other growth habits (i.e. non-arboreous) or generalist reproductive traits. Our predictions were that (*i*) areas with a high occurrence probability (>80%) of flowering plant endemic to the Caatinga will be reduced under two climate change scenarios: optimistic (moderate predicted–GHG emissions) and pessimistic (highest predicted GHG emissions), and (*ii*) the reduction of suitable habitats would be greatest for arboreous plants and those with specialized pollination systems, obligatory cross-pollination (self-incompatible/dioecious systems) and biotic dispersal modes.

## Material and methods

### Survey of Caatinga endemic plant species

A list of endemic Caatinga angiosperm species was initially generated from the Flora do Brasil website [57] using the following filters: "native of Brazil", "occurs only in the Northeast region", "occurs only in the phytogeographic domain of the Caatinga", "occurs only in the vegetation type of the Caatinga (*stricto sensu*)", thereby excluding rupestrian fields and altitude forest within the Caatinga, and "only endemic to Brazil". Using these search filters, 298 endemic species of angiosperms (now treated as plant species endemic to the Caatinga) were recorded.

**Fig 1:**
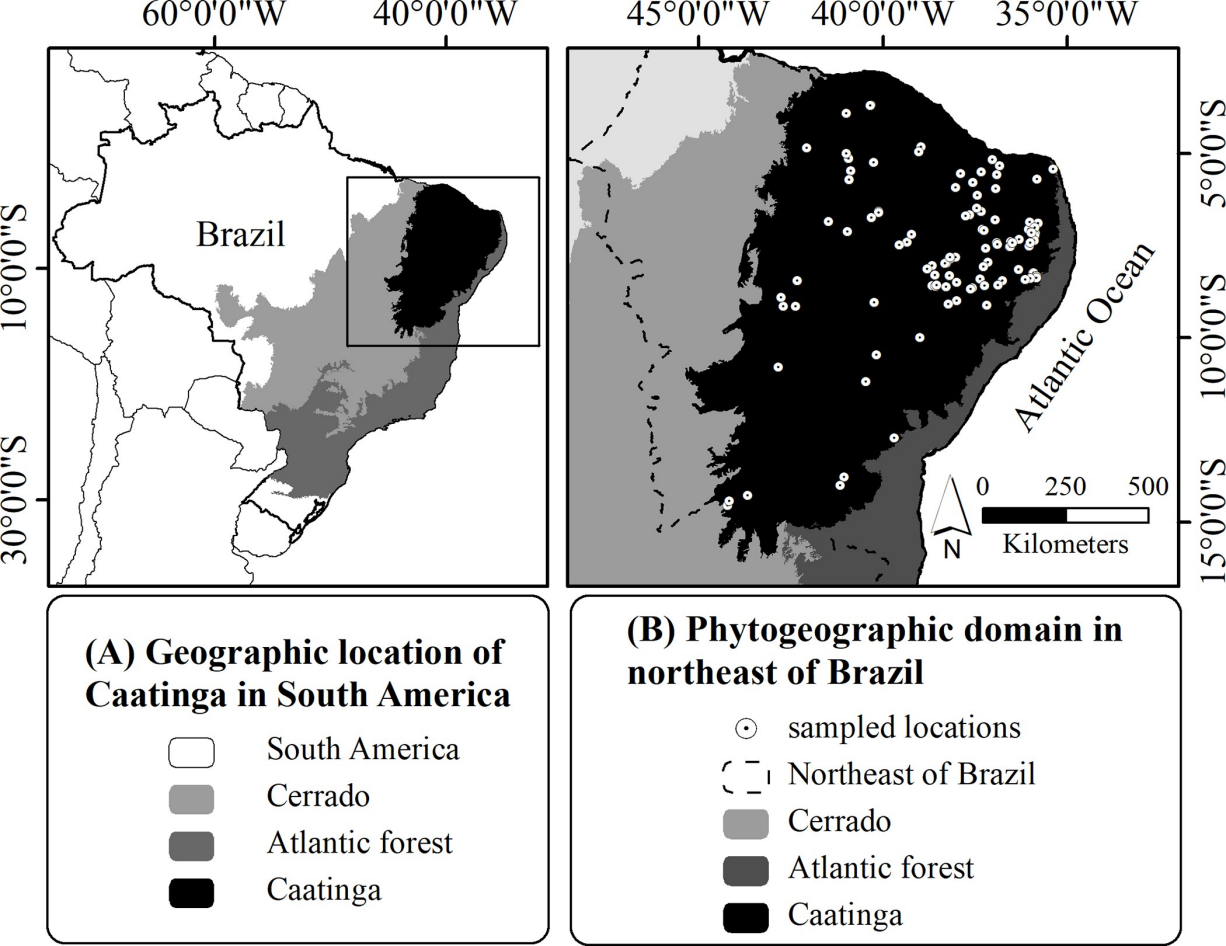
Location and geographic limits of the Caatinga. (A) Geographical location of the Caatinga in South America and (B) Phytogeographic domains in northeastern Brazil. Source of the shapes of the Brazilian phytogeographic domains: MMA-Ministério do Meio Ambiente, Brazil (public domain) (available for download at http://mapas.mma.gov.br/i3geo/datadownload.htm and http://mapas.mma.gov.br/mapas/aplic/probio/datadownload.htm?/caatinga/dados/shape_file/).

In order to test our hypotheses, we required georeferenced data (natural occurrence) of the plant species endemic to the Caatinga. Since biological collection networks such as SpeciesLink and GBIF provide georeferenced data without the possibility of filtering which points refer to individuals sampled in natural populations or cultivated in non-natural urban and rural areas, and we needed information exclusively from natural areas, we did not use georeferenced data from these databases. Alternatively, to assess precise information on the natural occurrence of plant species endemic to the Caatinga, we, therefore, used the catalogue of vascular plants by Moro *et al*. [54], which compiled this information for more than 2000 species from 98 studies. In addition, we obtained positional data for species from 16 other studies that had not been included in Moro *et al*. [54], because they are either more recent or non-floristic studies, but still contained checklists of Caatinga endemic plant species (S1 Table; Fig 1). The endemic species listed in these 114 studies had their synonymia and spelling checked based on the Flora do Brasil website [57]. In total, we obtained georeferenced data (132 points) for 76 out of the 298 plants species endemic to the Caatinga listed on the *Flora do Brasil* website [57]. We thus considered these 76 species in this study. The 132 geographic coordinates are distributed across the phytogeographic formations of Caatinga forest. The analyses were based on the occurrence of life history traits. S2 Table summarizes the number of species per georeferenced points and per life history strategy.

### Habit and reproductive trait characterization for Caatinga endemic species

All plant species were initially classified with respect to habit and reproductive traits. Species habit was obtained from: Moro *et al*. [54], the Flora do Brasil website and herbaria websites listed in The Plant List [60]. For the distribution modelling analysis, we grouped all species in each sample coordinate into two habit categories: (1) arboreous and (2) non-arboreous species (including other habits such as shrub, sub-shrub, herbaceous and scandent plants). Arboreous species were considered as a specialist habit due to growth and slow reproductive rate [39], being more vulnerable to environmental change.

For the reproductive trait characterization each species was classified into 41 categories of six major classes of “reproductive traits”: 1) floral biology: including flower type (*sensu* [33, 34, 61]), flower size (according to [62]) and floral reward (according to [61, 63, 64]), 2) pollination systems (according to [61, 63, 64] and to [65] for bee body size), 3) sexual systems, (according to [63, 64, 66]); 4) reproductive systems (according to [63, 64, 66, 67]), 5) fruit type (according to [68]), and 6) dispersal modes (according to [69]) (see Table 1 for details). For the ecological niche modeling analysis, from this initial classification into 41 reproductive trait categories, we regrouped species according to their degree of specialization (i.e., generalist *vs* specialist) into three more general biologically meaningful reproductive strategies (following [33]): 1) pollination system [generalist (e.g. small-sized bees, wasps, diverse small insects, butterflies, flies, moths, and wind) *vs*. specialized pollination systems (e.g. bats, medium-large bees, birds, beetles, Sphingids)] (*sensu* [33, 70]); 2) reproductive system [(generalist (e.g. self-compatible) *vs* specialized reproductive system (e.g. self-incompatible + dioecious (obligatory cross-pollinated species)]; 3) dispersal mode [generalist or abiotic (e.g. wind-dispersed and ballistic species) *vs* specialized or biotic dispersal mode (e.g. animal dispersed species)]. Reproductive traits, such as floral size, resource and sexual systems, were used to support the classification of pollination and reproductive systems, respectively.

Our hypotheses were based on this classification of generalization and specialization since this can elucidate significant diagnostics of species vulnerability to habitat changes mediated by human disturbances and climate change. Specifically, plant species bearing specialized reproductive strategies rely on the interactions with few species of pollinators or seed dispersers. Since habitat changes negatively affect populations of pollinators and seed dispersers, reproduction of plants with specialized reproductive strategies may be disproportionally affected in comparison to those with generalist strategies. The reproductive characterization of each species was based on: (1) botanical monographs and floras (e.g. [71–74]), (2) web searches, including published and referenced data, (3) field observations and a survey of specimens from the UFP Herbarium, and (4) our own personal observations and communications. Although a few tree species were incompletely assigned to all life-history trait categories, this unlikely introduced biases to the between-trait comparisons as all scenarios were compared in terms of the proportion of species within categories.

**Table 1:** Classes of reproductive life history traits with their respective categories used in this study.

Traits	Trait categories
Floral biology	
Flower type^1^	camera, tube, disc, bell/funnel, gullet, inconspicuous, brush, flag
Flower size^2^	inconspicuous (≤4 mm), small (>4 ≤10 mm), medium (>10 ≤20 mm), large (>20 ≤30 mm), very large (>30 mm)
Floral reward^3^	nectar, pollen, oil, resin
Pollination systems^3^	birds, bats, wind, small bees (< 12mm), medium-large bees (> = 12mm), diverse small insects (DSI), moths, Sphingids (hawkmoths), flies, beetles, butterflies
Sexual systems^4^	hermaphrodite, monoecious, dioecious
Reproductive systems^4^^,^^5^	self-compatible, self-incompatible, agamospermy, obligatory cross-pollination (self-incompatible species + dioecious)
Fruit type^6^	dry, fleshy
Dispersal mode^7^	zoochory, autochory, anemochory

^1^According to [33, 34, 61]

^2^According to [62]

^3^According to [61, 63, 64] and to [65] for bee body size

^4^According to [63, 64, 66]

^5^Outcrossing (i.e. obligatory cross-pollination) according to [67]

^6^According to [68]

^7^According to [69].

### Models and climatic variables

The current climate was described on the basis of ecological modeling data and the georeferencing system of Worldclim 2.0 for the period 1970–2000. Climatic projections for the future, between 2070 and 2100, were obtained from the IPCC5 database calibrated according to current climate data from Worldclim 2.0. Initially, we used a package of 19 bioclimatic variables for each period, which are derived from average monthly temperature and precipitation, thereby representing greater biological relevance for simulations of species distributions [75]. Climatic variables with fine-scale spatial resolution provide greater congruence for plant species with restricted distributions [76]. Estimates of the future distribution of Caatinga endemic plant species were based on the combination of global models of atmospheric and oceanic circulation, MIROC-ESM [77] and CCSM4 [78].

Climate models selected for the future in this study are projected as two Representative Concentration Pathways (RCPs) scenarios, which are based on GHG emissions and the trajectory that GHGs will present [1]. These models are inferred from aspects related to the carbon biogeochemical cycle, atmospheric and oceanic chemistry, vegetation types, emission of pollutants, solar radiation, ozone concentration, hydrology and sea ice [1]. In addition, the MIROC-ESM model includes simulations of ecological processes such as vegetation dynamics and terrestrial carbon cycling [77], while the CCSM4 model presents simulations for El Niño Southern Oscillation (ENSO) events [78]. For each model, two RCP climate change scenarios were selected. The RCP4.5 scenario is intermediate (considering the four available RCP scenarios: 2.6, 4.5, 6.0 e 8.5, whereby RCP2.6 is the more optimistic and RCP8.5 is the most pessimistic), in relation to predicted GHG emissions, i.e. predicting milder changes in temperature, such as an average increase of 1.8°C. We selected the RCP4.5 instead of the RCP2.6 since the latter is a stringent mitigation scenario, which is rarely considered in other studies (see also [29]). The second selected scenario (RCP8.5) is the most pessimistic, predicting the highest GHG emissions, and a mean increase in terrestrial temperatures of 3.7°C [1].

We selected bioclimatic layers with 30 arc-sec resolution grids (~1 km^2^) for both contemporary times and the two RCP scenarios. Bioclimatic variable data layers were cut to cover the entire extent of the Caatinga biome.

### Modeling potential life history trait distribution

After slicing bioclimatic layers, we performed correlation tests to exclude bioclimatic variables (r > 0.9) that were highly correlated either within or between the two climate models used (MIROC-ESM and CCSM4). Excluding strongly correlated variables ensures bioclimatic models with greater biological relevance for a regional set of species during the periods considered. In total, a set of eight bioclimatic variables were retained here: (1) isothermality (b3), (2) annual temperature range (b7), (3) mean temperature of warmest quarter (b10), (4) mean temperature of coldest quarter (b11), (5) precipitation seasonality (b15), (6) precipitation of driest quarter (b17), (7) precipitation of warmest quarter (b18) and (8) precipitation of coldest quarter (b19).

We used an algorithm based on the maximum entropy method to predict the most suitable habitat areas of plant species endemic to the Caatinga according to the reproductive traits considered using the Maxent v. 3.3 software [79]. The maximum entropy method implemented in Maxent is adequate for presence-only data, as in the localities sampled in this study. In addition, Maxent performs better compared to other software modeling species distributions, when it considers presence-only species distribution data [80].

The distribution data for each generalist or specialized life history strategy was divided into a training group (75% of the 132 sampled occurrence points) and a test or validation group (25% of the 132 sampled occurrence points) to calibrate, optimize and evaluate the quality of models generated. We used the AUC, area under the operator characteristic curve (ROC), as a measure of model ability to discriminate sites based on species presence and absence to estimate model quality. AUC values ranged from 0 to 1. In this sense, models best adjusted to the data have AUC values closer to 1. In addition, we used the gain values to estimate the proximity between models generated and the species incidence points for each reproductive trait sampled. Higher gain values indicate greater proximity between models and sampled points [79]. Modeling was performed 30 times for each generalist and specialized life history strategy under the current RCP4.5 and RCP8.5 scenarios and mean AUC and gain values were calculated (Table 2). We also considered the TSS (True Skill Statistics) value of the models (Table 2), which is a more realistic and practical method compared to AUC [(e.g. [81, 82]). TSS values range from -1 to 1, where 1 indicates perfect agreement and values of zero or negative indicate a performance no better than random [81].

**Table 2:** Adjustments between the sampled points of the endemic plant species in the Caatinga and climatic models for the current period (1970–2000) and two future scenarios (2070–2099), an optimist (RCP4.5) and a more pessimist (RCP8.5).

Traits	Current			RCP4.5			RCP8.5		
	AUC	Gain	TSS	AUC	Gain	TSS	AUC	Gain	TSS
	Mean±SD			Mean±SD			Mean±SD		
**Habit**									
Arboreous (N = 24)	0.98±0.005	2.78±0.01	0.7441	0.98±0.004	2.43±0.01	0.698	0.98±0.004	2.49±0.021	0.8333
Non-arboreous (N = 52)	0.95±0.013	2.65±0.18	0.6704	0.96±0.011	2.7±0.09	0.6158	0.96±0.01	2.68±0.03	0.6297
**Pollination systems**									
Specialized (N = 20)	0.97±0.009	2418±0.09	0.6417	0.97±0.008	2.53±0.2	0.5975	0.98±0.006	2.74±0.1	0.6842
Generalists (N = 30)	0.97±0.01	2.48±0.1	0.5577	0.97±0.011	2.7±0.101	0.660	0.97±0.009	2.64±0.08	0.6081
**Dispersal modes**									
Abiotic (N = 40)	0.96±0.009	2.34±0.3	0.5648	0.97±0.007	2.52±0.08	0.5203	0.98±0.005	2.65±0.03	0.6581
Biotic (N = 20)	0.97±0.009	2.7±0.1	0.7151	0.98±0.006	3.03±0.03	0.7185	0.98±0.005	3.05±0.02	0.7699
**Reproductive systems**									
Self-compatible (N = 9)	0.95±0.03	2.01±0.1	0.6855	0.96±0.021	2.29±0.1	0.6421	0.96±0.018	2.24±0.07	0.6023
Obligatory cross-pollination (self-incompatible+dioecious) (N = 12)	0.93±0.031	2.09±0.02	0.6497	0.96±0.014	2.46±0.09	0.6002	0.96±0.017	2.56±0.01	0.7089

AUC (area under operator curve) and gain for habit are represented by mean and standard deviation (SD) for each analyzed reproductive trait.

Based on the generated models, we constructed maps of the areas with different habitat probability of the species of each functional group. For analytical purposes, we defined suitable habitat areas those with a high probability of occurrence (>80%) for groups of endemic plant species with generalist or specialized reproductive strategies at each sample coordinate. The extent of suitable habitats was calculated separately for each trait. All modeling results were checked and edited using ArcGIS 10.0 software [83].

### Statistical analysis

To test whether suitable habitat areas for endemic species will be reduced by climate change, we compared the raw data among the current period and the two climate change scenarios using the one-way ANOVA and Kruskal-Wallis tests. Our second hypothesis—that reproductive trait specialization leads to greater vulnerability to climate-induced range contraction—was also tested using a two-way ANOVA and Kruskal-Wallis tests. Since the extent of suitable habitats under the RCP scenarios is related to the current extent for most traits considered (S3 Table), raw data were converted into percentage values to allow comparisons of suitable habitats between generalist and specialist traits among the current and future RCP scenarios. Specifically, we compared the extent of suitable habitats, based on percentage values, for trees and non-arboreous species, with either generalist or specialized pollination systems, abiotic or biotic dispersal modes, and self-compatible or obligatory cross-pollination (dioecious and self-incompatible reproductive systems). Tukey or Dunn posteriori tests were used to identify differences in the extent of suitable habitats for endemic species and their respective traits for all comparisons. We used Shapiro-Wilk and Bartlett tests, respectively, to check for normality of residuals and homogeneity of variances based on raw and percentage data in all cases. All analyses were performed within the R 3.3.1 environment [84].

## Results

**Fig 2:**
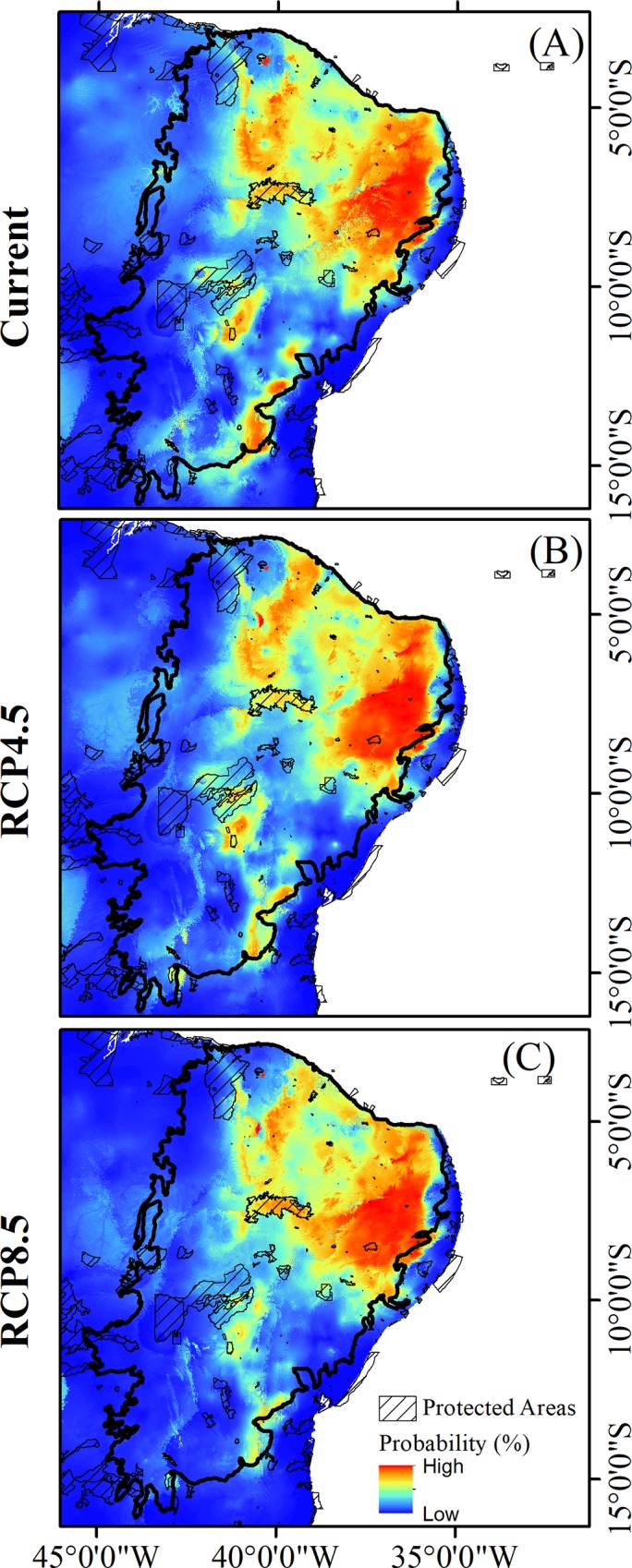
Distribution of suitable habitats of plant species endemic to the Caatinga. (A) During both the current period and two future scenarios, between 2070 and 2099, including a more optimistic (RCP4.5) (B) and a more pessimistic (RCP8.5) (C) projection. Solid dots in A indicate locations of the sampled species. Source of the shapes of the Brazilian and Caatinga boundaries, and protected areas: MMA-Ministério do Meio Ambiente, Brazil (public domain) (available for download, respectively, at http://mapas.mma.gov.br/i3geo/datadownload.htm; http://mapas.mma.gov.br/mapas/aplic/probio/datadownload.htm?/caatinga/dados/shape_file/); http://www.mma.gov.br/areas-protegidas/cadastro-nacional-de-ucs/dados-georreferenciados.html.

Compared to contemporary climate, suitable habitat areas for endemic plant species across the Caatinga biome will most likely be reduced in the future under both the optimistic and pessimistic climate change scenarios (F_2,87_ = 67.85; P<0.0001; Table 3; Fig 2), thereby corroborating our first hypothesis.

**Table 3:** Reductions in suitable habitats (i.e. areas with probability of occurrence > 80%) of endemic plant species in the Caatinga in two scenarios of climate change: An optimistic (RCP4.5) and a more pessimistic (RCP8.5).

	Suitable habitats (km^2^)						
	Current	RCP4.5		RCP8.5		Test	*P*
	Mean±SD	Mean±SD	% of loss or gain*	Mean±SD	% of loss or gain**		
Endemic plant species	122581.7±4877.9^a^	110788.1±4308.6^b^	↓9.62	110440.5±4583.7^b^	↓9.90	*F*_*2*,*87*_ = 67.85	0.001
**Habit**							
Arboreous	119288.3±7234.5^a^	106473.5±4773.1^b^	↓11.74	110094.3±7401.0^b^	↓7.71	*F*_*2*,*87*_ = 30.24	0.001
Non arboreous	112983.3±4530.0^a^	103499.3±3483.5^b^	↓8.39	98567.6±3571.8^c^	↓12.76	*F*_*2*,*87*_ = 106.4	0.001
**Pollination systems**							
Generalist	111537.6±6329.7^a^	107711.7±5013.4^b^	↓3.43	100276.9±4255.0^c^	↓10.09	*F*_*2*,*87*_ = 35.4	0.001
Specialized	107994.4±5434.7^a^	93500.4±2736.3^b^	↓13.42	94776.7±3025.6^b^	↓12.24	*F*_*2*,*87*_ = 125.5	0.001
**Dispersal modes**							
Abiotic	119282.0±5458.4^a^	99896.4±3716.0^c^	↓16.25	10338.3±3404.8^b^	↓8.67	*F*_*2*,*87*_ = 174.1	0.001
Biotic	84314.2±19416.0^a^	90201.4 ±17068.8^a^	↑6.98	93551.2±3677.1^a^	↑10.95	*F*_*2*,*87*_ = 2.89	0.07
**Reproductive systems**							
Self-compatible	168065.1±15429.1^a^	136967.9±17730.4^b^	↓18.50	133532.9±10519.9^b^	↓20.54	*H* = 47.955	0.001
Self-incompatible/ Dioecious	166369.3±7255.8^a^	127526.6±7038.0^b^	↓23.35	118489.1±4619.0^c^	↓28.78	*F*_*2*,*87*_ = 471.6	0.001

Significant differences in post-hoc comparisons among scenarios are indicated by different letters in the same row. Percentage of loss (↓) or gain (↑) of suitable habitat area comparing the current scenario with RCP4.5* and the current with RCP8.5**.

**Fig 3:**
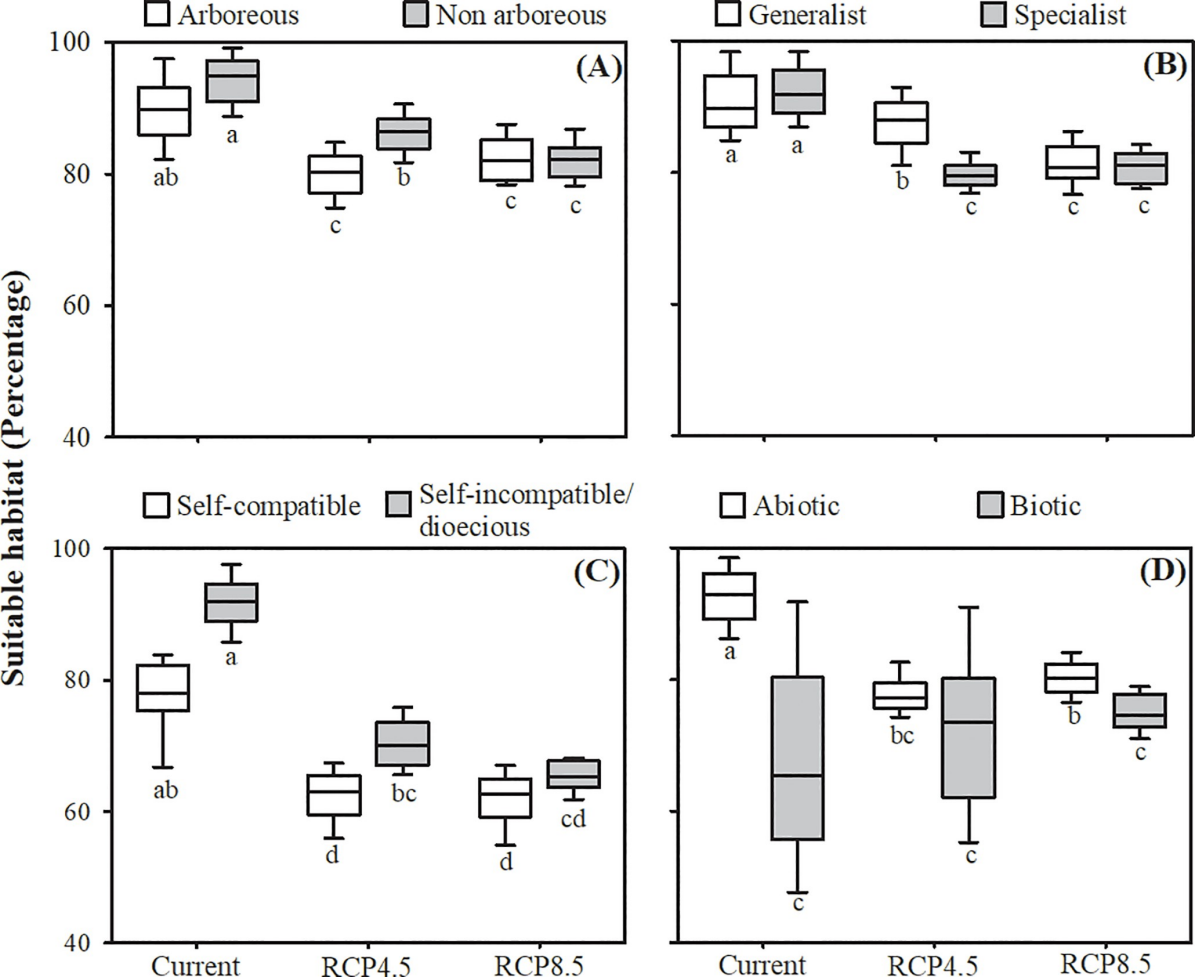
Reduction of suitable Caatinga habitat (probability of occurrence > 80%) for endemic flowering plant species in the current period and in two future scenarios, an optimistic (RCP4.5) and a pessimistic (RCP8.5). (A) Habit (N = 76). (B) Pollination systems (N = 64). (C) Reproductive systems (N = 25). (D) Dispersal modes (N = 62). Different letters below the boxplots indicate statistical significance of post-hoc comparisons at *P* < 0.05.

Compared to current times, the overall extent of suitable habitat will be significantly reduced for endemic species exhibiting any habit (by up to 12%), pollination system (up to 13%), reproductive system (up to 28%), and abiotic dispersal mode (up to 16%) under both climate change scenarios (Table 3; Fig 3A–3D). In terms of biotic dispersal modes, high occurrence probability areas are not predicted to be significantly altered for endemic plant species under any of the climate change scenarios considered here (Table 3; Fig 3D).

**Fig 4:**
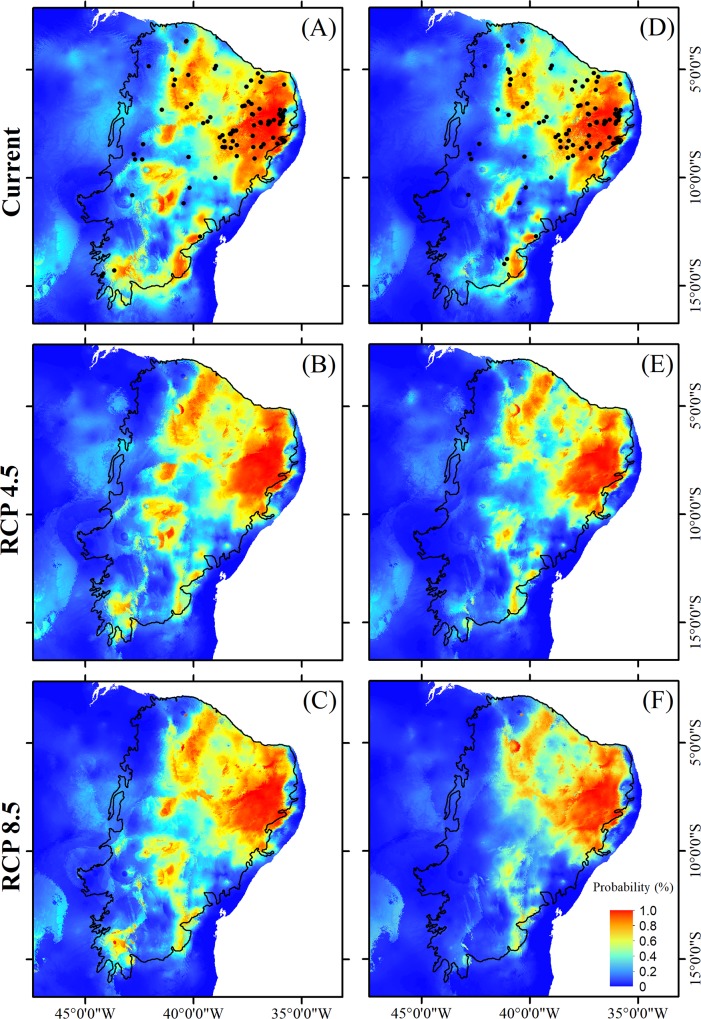
**Distribution of suitable habitat for arboreous species (A, B and C) and non-arboreous species (D, E and F) endemic to the Caatinga during both the present and two future scenarios, between 2070 and 2099, including an optimistic (RCP4.5) and a pessimistic (RCP8.5) scenario.** Solid dots in A and D indicate locations of the sampled species. Source of the shapes of the Brazilian and Caatinga boundaries: MMA-Ministério do Meio Ambiente, Brazil (public domain) (available for download at http://mapas.mma.gov.br/i3geo/datadownload.htm and http://mapas.mma.gov.br/mapas/aplic/probio/datadownload.htm?/caatinga/dados/shape_file/).

**Fig 5:**
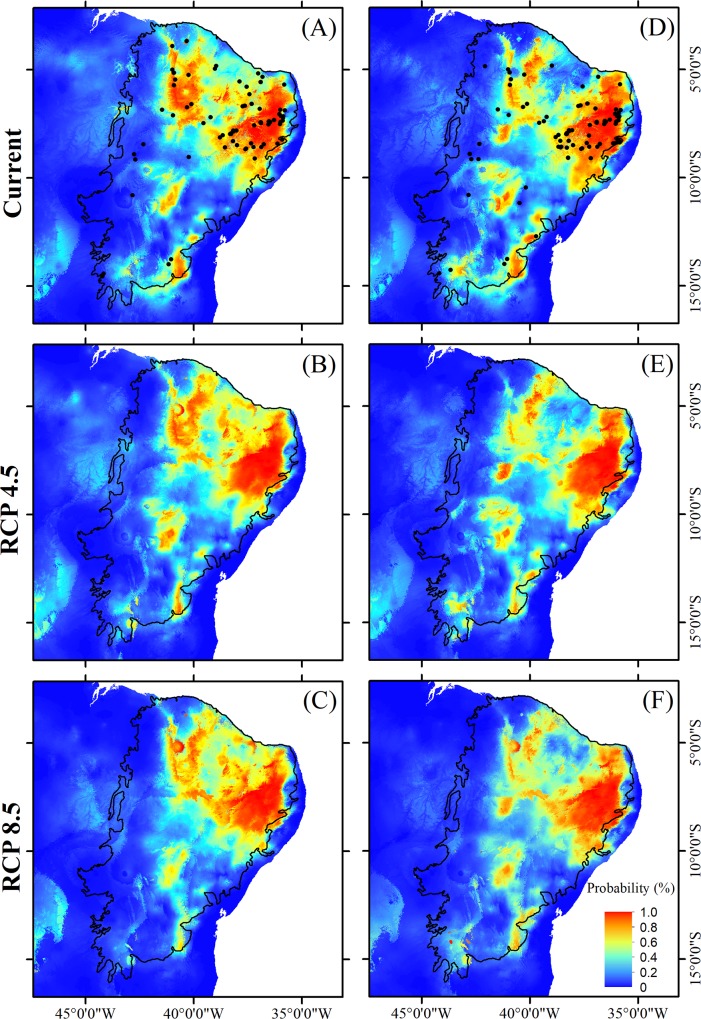
**Distribution of suitable habitat for plant species endemic to the Caatinga with either generalist (A, B and C) or specialized (D, E and F) pollination systems in both the present and two futures scenarios, between 2070 and 2099, including an optimistic (RCP4.5) and a pessimistic (RCP8.5) scenario.** Solid dots in A and D indicate locations of the sampled species. Source of the shapes of the Brazilian and Caatinga boundaries: MMA-Ministério do Meio Ambiente, Brazil (public domain) (available for download at http://mapas.mma.gov.br/i3geo/datadownload.htm and http://mapas.mma.gov.br/mapas/aplic/probio/datadownload.htm?/caatinga/dados/shape_file/).

**Fig 6:**
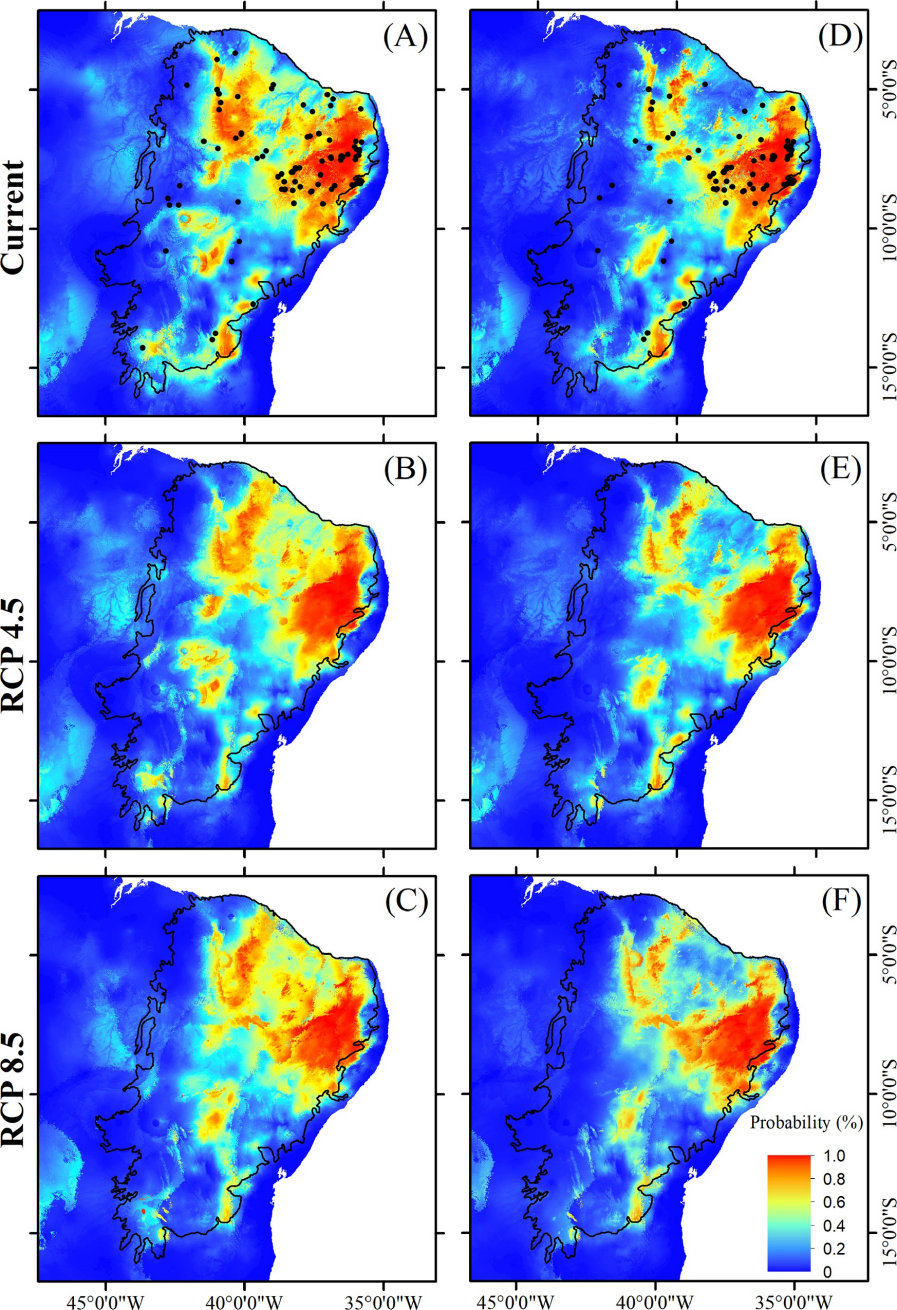
**Distribution of suitable habitat for plants species endemic to the Caatinga with either self-compatible (A, B and C) or self-incompatible/dioecious (i.e. obligatory cross-pollination) (D, E and F) reproductive systems in the present and two future scenarios, between 2070 and 2099, including an optimistic (RCP4.5) and a pessimistic (RCP8.5).** Solid dots in A and D indicate locations of the sampled species. Source of the shapes of the Brazilian and Caatinga boundaries: MMA-Ministério do Meio Ambiente, Brazil (public domain) (available for download at http://mapas.mma.gov.br/i3geo/datadownload.htm and http://mapas.mma.gov.br/mapas/aplic/probio/datadownload.htm?/caatinga/dados/shape_file/).

**Fig 7:**
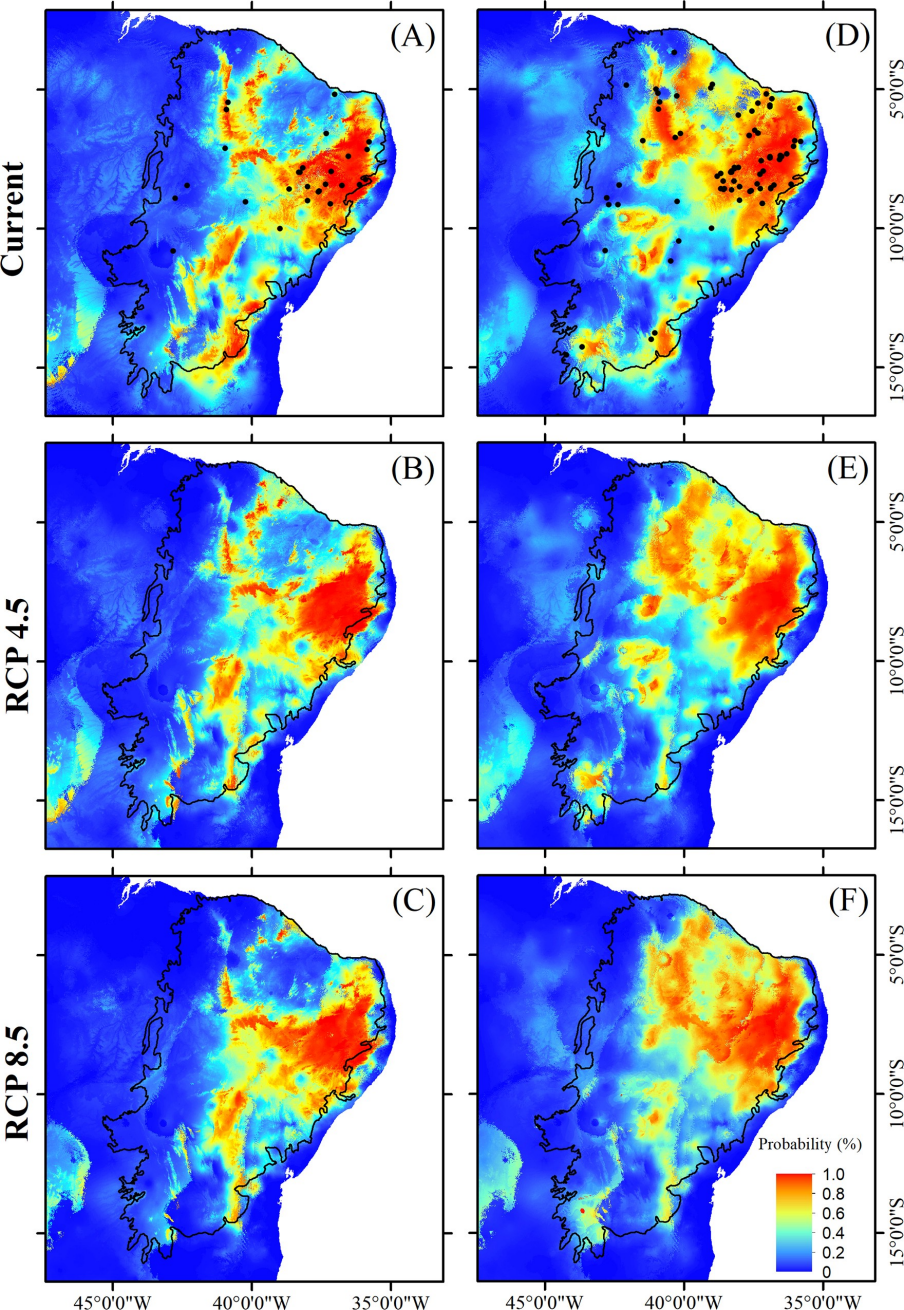
**Distribution of suitable habitat for plant species endemic to the Caatinga with either abiotic (A, B and C) or biotic (D, E and F) seed dispersal modes in both the present and two futures scenarios between 2070 and 2099, including an optimistic (RCP4.5) and a pessimistic (RCP8.5).** Solid dots in A and D indicate locations of the sampled species. Source of the shapes of the Brazilian and Caatinga boundaries: MMA-Ministério do Meio Ambiente, Brazil (public domain) (available for download at http://mapas.mma.gov.br/i3geo/datadownload.htm and http://mapas.mma.gov.br/mapas/aplic/probio/datadownload.htm?/caatinga/dados/shape_file/).

Comparing the two climate change scenarios separately, divergent responses were observed for different plant traits. In relation to the current scenario, areas with a high occurrence probability for tree species (Fig 4D–4F), species with specialized pollination systems (Fig 5D–5F), and those with self-compatible reproductive systems (Fig 6A–6C) tend to be similarly reduced under both the best-case and worst-case scenarios. In the case of non-arboreous species (Fig 4A–4C), species with generalist pollination systems (Fig 5A–5C), and obligatory cross-pollinated species (Fig 6D–6F), reduced areas of high occurrence probability will be even more severe under the pessimistic scenario (Table 3; Fig 3). Conversely, suitable habitats for species with abiotic dispersal modes will be most reduced under the optimistic climate change scenario (Table 3; Fig 7).

Considering plant habits, both tree and non-arboreous species were reduced in their future extent of occurrence compared to present times. However, reductions in high occurrence probability areas were most severe for non-arboreous species, compared to tree species, under both climate change scenarios (Fig 3A). This reduction was greater under the pessimistic scenario of climate change (H = 107.15, df = 5; P = 0.0001; Fig 3A).

Reductions in suitable habitat areas were greater for species with specialized reproductive traits, compared to generalist species, thereby corroborating our second hypothesis (pollination systems: F_2,174_ = 23.89; P < 0.0001; reproductive systems: H = 130.46; P < 0.0001; Fig 3B and 3C). On the other hand, our second hypothesis was only partly corroborated for species with distinct dispersal modes. Reduced suitable habitats was observed for only those species with abiotic dispersal modes. However, when compared to species with biotic dispersal, species with abiotic dispersal modes retained larger areas of suitable habitats under both the current and future (RCP4.5 and RCP8.5) scenarios (H = 87.421; df = 5; P < 0.0001; Fig 3D).

## Discussion

Our results indicate that, despite drastic predicted changes in climatic conditions, suitable habitats for plant species endemic to the Caatinga semi-arid region will tend to remain within this phytogeographic domain boundaries. Specifically, regardless of the intensity of climate change projections examined here, suitable habitat areas for endemic plant species—with any of the life-history traits we considered—will most likely be reduced and concentrated in the northeasternmost portion of the Caatinga (Figs 4–7), which is nearest the coast, and therefore least arid. In addition, suitable habitats for species with specialized reproductive strategies, such as obligatory cross-pollination, will be generally reduced more intensely (up to 28%) compared to the analogous areas hosting species with generalist reproductive strategies (Fig 3). Above and beyond climatic conditions, the long-term viability of plants endemic to the Caatinga will also rely on the persistence of suitable habitat remnants. The Caatinga vegetation is currently reduced to 50% of its original extension due to expansion and intensification of anthropogenic activities. In addition, the remaining Caatinga vegetation, is now distributed across ca. 41,700 remnants mainly represented by forests patches smaller than 500 ha [85]. These small forest patches are concentrated in the eastern portion of the Caatinga, which according to our results, coincides with areas of suitable climatic conditions for endemic plant species, in both the current and future scenarios. The synergistic combination of climate change and Caatinga habitat loss through rapidly escalating land-use can potentially exacerbate the loss of suitable habitat for endemic plant species, and this is even more intensive for species with specialized reproductive traits. Several key ecological interactions, plant community dynamics, and the maintenance of native biodiversity are therefore particularly threatened in this SDTF, as discussed below.

Our findings support the hypothesis that plant species endemic to the Caatinga, especially those with specialized reproductive traits, are vulnerable to the effects of climate change, because their suitable habitat area will be reduced in both scenarios of climate projections. Geographic range contraction for endemic flowering plant species in seasonally-dry tropical forests have been predicted or observed previously (e.g. [6, 86–88]), which is further reinforced by our results considering growth habits and reproductive functional traits. However, suitable habitat fidelity for Caatinga endemic plants is consistent with predictions for plants and birds endemics to tropical dry forests of Mexico and/or Mesoamerica, which indicate their future persistence in their current domains [89].

The concentration of habitats with suitable climatic conditions for endemic plant species in the northeastern most and least arid portion of this biome may be explained by abiotic factors, such as rainfall and water sources. Indeed, the plant species composition in the Caatinga may be strongly associated with rainfall (e.g. [54, 55]). In addition to increased precipitation in the peripheral regions compared to the core of Caatinga, the large concentration of rivers in the northern portion of this biome [86] may be positively associated with the distribution of habitats with suitable climatic conditions for endemic plants. This likely maintenance of suitable habitats for endemic plant species suggests habitat probable under climatic conditions reversing increased aridity. Coastal areas ensure higher humidity from the Atlantic-Equatorial air masses, which may result in greater water soil availability (see [90]). Notwithstanding this concentration in the most favorable portion of the Caatinga, suitable climatic conditions for endemic plant species are unlikely to be displaced to areas outside their present phytogeographic domain even under severe climate change. All native plant species in the Caatinga, whether or not they are endemic, present a set of morphological, anatomical and ecophysiological adaptations to arid conditions, which ensures their permanence in this domain even under conditions of elevated temperatures and lower rainfall [91]. Among these traits, we highlight leaf abscission during the dry season, xylem that tolerates high negative pressures, high water storage, and high accumulation of epicuticular wax ([91] and references therein).

As a consequence of shifts in the distribution of suitable climatic conditions for endemic plant species with the reproductive strategies considered here, negative cascade effects on plant-animal interactions, including pollinators and seed dispersers, are expected. With hotter and drier conditions, plant-pollinator interactions may be disrupted by advances or delays in flowering and fruiting phenology [12, 13, 92, 93]. In parallel, some groups of pollinators and seed dispersers may face reduced suitable habitat and changes in pupation and emergence times (e.g. [38, 94–97]). The combination of these changes may lead to a reciprocal phenological asynchrony, which would affect reproductive events of both plants and their animal mutualists [98, 99]. For instance, interactions between specific pollinators and their respective mutualistic plant species can be disrupted and even extirpated at local scales [12].

Endemic species are usually associated with restricted ranges, small populations, and specialized habitat requirements (with narrow spectrum of conditions and adequate environmental resources) [43]. In a global study on extinction risk in endemic species within biodiversity hotspots, habitat specificity was, the most influential variable in the potential loss of 56,000 plant species and 3,700 vertebrate species worldwide [44]. In addition to these habitat requirements, the disruption of ecological interactions, such as pollination and seed dispersal, may also be associated with the extinction of endemic plant species, especially those with specialized reproductive strategies [33]. Indeed, plant species that strictly depend on pollinators [obligatory cross-pollination (self-incompatible + dioecious) like as *Apterokarpus gardneri*, Anacardiaceae], which are pollinated by a single pollination vector or by a reduced group of specific pollinators such as bats (*Calliandra aeschynomenoides*, Fabaceae), Sphingid moths, and non-flying vertebrates are more prone to the disruption of these ecological interactions mainly in human modified landscapes in tropical regions (e.g. [33, 100]). These negative effects of climate change on the distribution of endemic plant species may be intensified due to anthropogenic disturbances. For example, SDTFs have been the historically most preferred regions for agricultural development and human settlements in Meso and South America [101–104]. In Brazil, the Caatinga domain is mainly inhabited by the rural poor who continue to intensively extract forest resources to meet their basic livelihoods [85, 105]. Considering forage potential, *Libidibia ferrea* (jucá) and fruits of *Spondias tuberosa* (umbuzeiro) are used as feed for goats, sheep and cattle [88]. Some species such as *Ziziphus joazeiro* (Juazeiro), *Poincianella microphylla* and *Poincianella pyramidalis* are exploited by the population to produce firewood, charcoal and / or cuttings without any cultivation technique [106]. *Poincianella pyramidalis* is commonly used in folk medicine [107]. Moreover, less than 2% of the Caatinga biome is strictly protected [108]. Alarmingly, when we overlapped our suitable habitat projections for endemic plant species with the distribution of all protected areas in the Caatinga, we observed few conservation units covering future potential distribution areas, further aggravating the vulnerability of these species (Fig 2). This in turn may be particularly relevant to the productivity and resilience of the Caatinga [109]. Anthropogenic activities create barriers that hinder or preclude species movements [110]. Therefore, the synergistic interaction between climate change and acute or chronic anthropogenic habitat disturbance threatens the persistence, dynamics and functioning of dry tropical forest ecosystems, and their biodiversity (e.g. [102, 111–113]).

## Conclusion

In conclusion, plant species endemic to the Caatinga are highly vulnerable to even conservative scenarios of future climate change and may lose much of their climatic envelopes. These threats are even greater for endemic species with specialized reproductive traits. Consequently, by reducing areas of suitable climatic conditions, climate change may disrupt key ecological interactions, such as pollination and seed dispersal, and compromise species maintenance and dynamics of communities of plants and animals in the Caatinga. The Caatinga has been historically threatened by chronic anthropogenic disturbances and the effects of climate change will further aggravate the impacts of these disturbances on biodiversity, particularly endemic plants. Studies investigating biodiversity and functional trait responses to the synergistic effects of both climate and land-use change are extremely important to guide management plans, and the conservation of natural resources in the Caatinga. Furthermore, since the Caatinga remains the least protected of all major biomes in Brazil, we suggest the creation of new protected areas that consider both the present and future species occurrence. New protected areas should, therefore, be located in the northeasternmost portion of the Caatinga, which has a more favorable climate, but is currently exposed to escalating agricultural intensification.

## Supporting information

S1 Table Complementary papers with geographic coordinates of the plant species endemic to the Caatinga. (PDF)

S2 Table Number of endemic plant species to Caatinga dry forest per georeferenced point and per trait. (PDF)

S3 Table Relationship between the current and future suitable habitat across the Caatinga domain (i.e. probability of occurrence > 80%) for endemic flowering plants. Comparisons were performed separately for each category within habit, pollination systems, reproductive systems and seed dispersal modes. Statistical significance was assessed by *P* < 0.05.
(PDF)
