# Supplementary material for: Climate change will reduce suitable Caatinga dry forest habitat for endemic plants with disproportionate impacts on specialized reproductive strategies
Source: PLoS One. 2019 May 29;14(5):e0217028. doi: 10.1371/journal.pone.0217028 (PMC6541362; doi:10.1371/journal.pone.0217028)
Supplement: S1 Table — (PDF) [file pone.0217028.s001.pdf]

N	Title	Authors	Year	Journal	Vol.	Pages
1	Regeneração natural em um remanescente de Caatinga sob diferentes níveis de perturbação, no agreste paraibano	Pereira IM, Andrade LA, Costa JRM, Dias JM	2001	Acta Botanica Brasilica	15 (3)	413-426
2	Composição florística e análise fitossociológica do componente arbustivo-arbóreo de um remanescente florestal no agreste paraibano	Pereira IM, Andrade LA, Barbosa MRV, Sampaio EVSB	2002	Acta Botanica Brasilica	16 (3)	357-369
3	Use-history effects on structure and flora of Caatinga	Pereira IM, Andrade LA, Sampaio EVSB, Barbosa MRV	2003	Biotropica	35 (2)	154-165
4	Floral traits and pollination systems in the Caatinga, a Brazilian Tropical Dry Forest	Machado IC & Lopes AV	2004	Annals of Botany	94	365-376
5	Plant sexual systems and a review of the breeding system Studies in the Caatinga, a Brazilian Tropical Dry Forest	Machado IC, Lopes AV & Sazima M	2006	Annals of Botany	97	277-287
6	Diversidade e estrutura fitossociológica da Caatinga na Estação Ecológica do Seridó-RN	Santana JAS & Souto JS	2006	Revista de Biologia e Ciências da Terra	6 (2)	232-242
7	Effects grazing by steers and a long drought on a caatinga ligneous stratum in semi-arid northeast, Brazil	Albuquerque SG, Soares JGG & Filho CG	2008	Revista Caatinga	21 (4)	17-28
8	Sítios de estabelecimento e relações alométricas em populações lenhosas da Caatinga	Figueiredo LS, Ferraz EMN, Rodal MJN, Pimentel RMM, Araújo EL	2010	Revista de Geografia	27 (2)	155-167
9	Levantamento florístico e fitossociológico em fragmentos de Caatinga no Município de Taboleiro Grande-RN	Bessa MAP & Medeiros JF	2011	GeoTemas	1 (2)	69-83
10	Angiosperms from the Araripe National Forest, Ceará, Brazil	Ribeiro-Silva S, Medeiros MB, Gomes BM, Seixas ENC, Silva MAP	2012	Checklist	8 (4)	744-751
11	Composição florística da RPPN Fazenda Almas, no Cariri Paraibano, Paraíba, Brasil	Lima IB & Barbosa MRV	2014	Revista Nordestina de Biologia	23 (1)	49-67
12	Flora vascular de um inselbergue na mesorregião do sertão paraibano, nordeste do Brasil	Lucena DS, Lucena MFA, Souza JM, Silva RFL, Souza PF	2014	Scientia Plena	11 (1)	1-11
13	Levantamento florístico e fitossociológico em área de Caatinga manipulada durante o período chuvoso	Luna AA, Carneiro MSS, Furtado RN, Silva GJGM, Camapanha MM, Medeiros HR	2015	Revista Científica de Produção Animal	17 (1)	41-49
14	Levantamento florístico de um trecho de mata ciliar na mesorregião do Sertão Paraibano	Silva FG, Silva RH, Araújo RM, Araújo MF, Sousa JM	2015	Revista Brasileira de Botânica	13 (4)	250-258
15	Estrutura da vegetação em dois fragmentos de Caatinga Antropizada na Paraíba	Sabino FGS, Cunha MCL, Santana GM	2016	Floresta e Ambiente	23 (4)	487-497
16	Levantamento florístico de um trecho de mata ciliar na mesorregião do Sertão Paraibano	Santos WS, Souza MP, Nóbrega GFQ, Medeiros, FS, Alves AR, Holanda AC	2017	Revista Nativa	5 (2)	85-91
