# Supplementary material for: Climate change will reduce suitable Caatinga dry forest habitat for endemic plants with disproportionate impacts on specialized reproductive strategies
Source: PLoS One. 2019 May 29;14(5):e0217028. doi: 10.1371/journal.pone.0217028 (PMC6541362; doi:10.1371/journal.pone.0217028)
Supplement: S2 Table — (PDF) [file pone.0217028.s002.pdf]

Table S2. Number of endemic plant species to Caatinga dry forest per georeferenced point and per trait.

Point	Coordinates		Total species	Traits							
				Habit		Pollination systems		Reproductive systems		Dispersal modes	
	Lat	Long		Arboreous	Non-arboreus	Generalists	Specialized	Self-compatible	Self-incompatible/ Dioecious	Biotic	Abiotic
**1**	-9	-38	8	4	4	1	6	1	2	3	4
**2**	-10	-39	5	3	2	1	4	1	3	2	3
**3**	-8,5	-38	5	3	2	1	4	3	1	3	2
**4**	-12,7167	-39,7	4	3	1	1	2	0	2	1	3
**5**	-12,6167	-38,2833	1	1	0	0	1	0	1	0	1
**6**	-7,11667	-40,9667	7	0	7	4	3	1	0	2	5
**7**	-5,46667	-40,8667	5	1	4	3	1	1	2	1	3
**8**	-5,71667	-40,9167	5	1	4	3	1	1	2	1	3
**9**	-7,81667	-38,0333	5	1	4	3	2	1	1	3	2
**10**	-7,86667	-38,1833	5	1	4	3	2	1	1	3	2
**11**	-7,95	-38,2833	5	2	3	2	2	1	2	1	4
**12**	-7,98333	-38,3167	5	2	3	2	2	1	2	1	4
**13**	-9,11667	-37,1833	3	3	0	1	2	0	2	0	2
**14**	-8,65	-37,5833	14	6	8	4	10	3	5	3	14
**15**	-8,68333	-37,6167	14	6	8	4	10	3	5	3	14
**16**	-13,7667	-41,05	6	5	1	2	2	0	1	1	5
**17**	-14	-41,1667	6	5	1	2	2	0	1	1	5
**18**	-3,90944	-40,99	1	0	1	1	0	1	0	0	1
**19**	-8,40306	-37,3583	7	4	3	0	7	1	4	2	5
**20**	-6,88111	-35,7947	4	2	2	2	2	2	1	1	3
**21**	-6,88111	-35,795	6	3	3	3	3	2	2	2	4

**22**	-8,44722	-42,3297	2	1	1	1	1	0	0	0	2
**23**	-8,90639	-42,7642	2	1	1	1	1	0	0	0	2
**24**	-9,15	-42,7	3	3	0	2	1	0	1	0	3
**25**	-8,23833	-35,9222	4	2	2	2	2	2	1	1	3
**26**	-7,81667	-38,1833	2	0	2	2	0	1	0	1	1
**27**	-7,98333	-38,2833	6	4	2	1	4	2	3	3	3
**28**	-7,16444	-35,8603	2	1	1	1	1	1	0	1	1
**29**	-5,17	-37,0294	1	0	1	1	0	1	0	0	1
**30**	-9,03333	-40,2333	3	1	2	2	1	2	1	2	1
**31**	-6,88944	-35,7986	6	3	3	3	3	2	2	2	4
**32**	-9,11667	-37,1833	5	3	2	2	3	1	3	2	3
**33**	-4,85	-42,0667	2	0	2	1	1	0	1	0	2
**34**	-8,44722	-42,3297	10	4	6	4	6	3	3	4	5
**35**	-8,90639	-42,7642	10	4	6	4	6	3	3	4	5
**36**	-8,45	-36,7667	10	5	5	2	7	4	4	4	4
**37**	-7,98333	-38,3167	10	5	5	2	7	4	4	4	4
**38**	-8,31194	-38,1958	1	0	1	1	0	0	0	0	1
**39**	-10,8	-42,8333	8	3	5	5	3	2	1	2	4
**40**	-7,4	-36,5333	2	1	1	1	1	1	1	0	2
**41**	-6,58333	-37,3333	2	1	1	1	1	1	1	0	2
**42**	-6,66667	-37,65	1	1	0	0	1	0	1	0	1
**43**	-5,17	-37,0294	1	1	0	0	1	0	1	0	1
**44**	-8,58333	-38,6667	1	1	0	0	1	1	0	1	0
**45**	-8,45	-36,7667	4	2	2	1	3	1	2	2	1
**46**	-8,58333	-37,25	12	7	5	3	9	2	2	3	8
**47**	-8,42639	-36,135	3	1	2	2	1	1	1	0	3

**48**	-8,23333	-35,9167	1	0	1	0	0	0	0	1	0
**49**	-7,9525	-37,1519	3	2	1	1	1	2	0	2	1
**50**	-6,58333	-37,3333	4	2	2	2	2	1	2	0	4
**51**	-6,66667	-37,65	4	2	2	2	2	1	2	0	4
**52**	-5,95167	-36,9447	1	0	1	1	0	0	0	0	1
**53**	-7,05267	-36,0517	6	3	3	3	3	2	2	3	3
**54**	-7,47083	-36,8975	3	1	2	1	2	1	1	1	2
**55**	-7,39667	-36,5319	8	4	4	3	4	2	3	2	6
**56**	-5,79278	-37,5569	2	1	1	1	1	0	0	1	1
**57**	-4,82611	-38,9692	1	0	1	0	1	0	0	0	1
**58**	-7,40694	-39,3497	10	4	6	6	4	3	2	1	9
**59**	-7,4	-36,5333	2	1	1	1	1	0	1	0	2
**60**	14,2758	-43,6656	1	1	0	0	1	0	1	0	1
**61**	-6,57361	-40,1236	2	0	2	2	0	1	0	0	2
**62**	-6,81	-36,9606	4	2	2	3	1	1	1	0	4
**63**	-6,3133	-37,433	3	1	2	2	1	1	1	0	3
**64**	-6,85861	-36,0153	8	5	3	4	3	2	2	3	5
**65**	-7,5725	-37,2192	8	5	3	4	3	2	2	3	5
**66**	-5,57222	-36,9092	2	0	2	2	0	1	0	0	2
**67**	-5,7925	-37,5567	3	1	2	2	1	1	1	0	3
**68**	-4,95556	-39,0244	2	1	1	2	0	1	1	0	2
**69**	-7,01689	-35,8806	1	1	0	0	1	1	0	1	0
**70**	-8,31194	-38,1958	6	5	1	1	4	1	3	3	3
**71**	-8,61667	-38,2833	4	1	3	2	2	1	2	1	3
**72**	-8,3	-38,5833	4	1	3	2	2	1	2	1	3
**73**	-7,81667	-38,1833	2	1	1	1	1	2	0	1	1

**74**	-5,55389	-37,8886	2	0	2	2	0	1	0	0	2
**75**	-14,5522	-44,2175	1	1	0	1	0	0	0	0	1
**76**	-14,5522	-44,2175	5	3	2	3	2	2	1	1	4
**77**	-5,69389	-35,8197	4	3	1	1	3	2	2	1	3
**78**	-8,33333	-35,8333	4	1	3	2	1	2	1	1	3
**79**	-7,33333	-36,3	4	1	3	3	1	2	1	0	4
**80**	-7,39167	-36,533	6	3	3	3	2	3	1	2	4
**81**	-8,15	-36,3208	1	0	1	1	0	0	0	0	0
**82**	-6,58333	-37,3333	4	2	2	2	2	1	2	0	4
**83**	-6,66667	-37,65	4	2	2	2	2	1	2	0	4
**84**	-8,60083	-38,5678	5	2	3	0	4	1	3	2	3
**85**	-9,0825	-38,2297	3	0	3	1	1	1	0	1	2
**86**	-9,09083	-38,2286	3	0	3	1	1	1	0	1	2
**87**	-8,3125	-38,1953	6	4	2	3	3	2	2	1	5
**88**	-11,1856	-40,4683	2	2	0	0	1	0	1	1	1
**89**	-10,4667	-40,1833	2	1	1	1	1	0	1	0	2
**90**	-7,39167	-36,5331	2	1	1	1	1	1	1	1	1
**91**	-5,34083	-36,8328	4	2	2	2	2	1	2	0	4
**92**	-6,70278	-37,7542	6	3	3	3	3	2	2	2	4
**93**	-8,06667	-37,2667	7	4	3	2	5	2	3	2	5
**94**	-8,38194	-35,8439	5	0	5	2	2	2	0	1	3
**95**	-8,39139	-36,0103	5	0	5	2	2	2	0	1	3
**96**	-7,43694	-36,9083	5	3	2	3	1	2	0	2	3
**97**	-7,42944	-36,9097	5	3	2	3	1	2	0	2	3
**98**	-7,53139	-36,5608	5	2	3	3	2	2	1	2	3
**99**	-7,52722	-36,5517	5	2	3	3	2	2	1	2	3

**100**	-7,42583	-36,4892	6	3	3	4	1	3	0	2	4
**101**	-7,42083	-36,4881	6	3	3	4	1	3	0	2	4
**102**	-6,60028	-40,1208	9	2	7	5	4	3	2	1	8
**103**	-6,74306	-40,3167	9	2	7	5	4	3	2	1	8
**104**	-6,85361	-41,4708	10	5	5	4	6	1	3	2	8
**105**	-8,03556	-38,6667	9	4	5	3	6	3	2	4	5
**106**	-8,13333	-38,8	9	4	5	3	6	3	2	4	5
**107**	-8,42639	-36,135	5	3	2	1	4	2	2	2	3
**108**	-8,57389	-38,5458	3	2	1	0	3	2	1	1	1
**109**	-7,23083	-35,8808	4	2	2	2	2	2	1	1	3
**110**	-7,35778	-35,8978	4	2	2	2	2	2	1	1	3
**111**	-7,52	-36,0269	4	2	2	2	2	2	1	1	3
**112**	-7,42	-35,9997	4	2	2	2	2	2	1	1	3
**113**	-5,14557	-40,9278	3	0	3	2	1	1	1	0	2
**114**	-5,1455	-40,9278	3	0	3	2	1	1	1	0	2
**115**	-5,14556	-40,283	3	0	3	2	1	1	1	0	2
**116**	-5,25	-40,25	22	5	17	12	7	8	6	3	15
**117**	-5	-41	22	5	17	12	7	8	6	3	15
**118**	-9,15	-42,3667	2	2	0	0	2	0	2	0	2
**119**	-3,68333	-40,3333	5	4	1	3	2	2	2	4	1
**120**	-8,575	-36,8792	7	4	3	1	5	2	3	3	3
**121**	-14,4244	-44,1719	3	1	2	2	1	1	1	0	1
**122**	-7,15058	-35,9754	4	1	3	3	1	0	2	2	2
**123**	-5,93	-38,0344	6	3	3	3	3	3	2	1	5
**124**	-7,195	-39,2244	3	1	2	2	1	2	0	2	0
**125**	-7,47722	-39,56	3	1	2	2	1	2	0	2	0

**126**	-5,42611	-35,3836	2	2	0	0	2	1	1	1	1
**127**	-7,47083	-36,8975	16	7	9	6	9	5	5	6	10
**128**	-7,06194	-37,2997	7	3	4	4	3	3	1	1	5
**129**	-3,7	-40,35	2	2	0	1	1	0	2	0	2
**130**	-6,47917	-37,4561	2	2	0	0	2	1	1	1	1
**131**	-7,08278	-37,2603	3	2	1	1	2	2	1	1	2
**132**	-5,49944	-37,3411	5	3	2	3	2	1	3	0	5
