# Supplementary material for: Climate change will reduce suitable Caatinga dry forest habitat for endemic plants with disproportionate impacts on specialized reproductive strategies
Source: PLoS One. 2019 May 29;14(5):e0217028. doi: 10.1371/journal.pone.0217028 (PMC6541362; doi:10.1371/journal.pone.0217028)
Supplement: S3 Table — Comparisons were performed separately for each category within habit, pollination systems, reproductive systems and seed dispersal modes. Statistical significance was assessed by P < 0.05. (PDF) [file pone.0217028.s003.pdf]

	Current v. RCP 4.5			Current v. RCP 8.5		
	Adjusted R^2	*t* value	*P*	Adjusted R^2	*t* value	*P*
**Endemic**	0.03164	1.396	0.17	-0.276	0.469	0.64
**Habit**						
Arboreous	0.2602	3.346	0.00	0.1749	2.673	0.01
Non-arboreous	0.4038	4.543	0.00	0.6497	7.402	0.00
**Pollination system**						
Generalist	0.6023	6.702	0.00	0.8491	12.813	0.00
Specialist	0.2134	2.978	0.00	0.6483	7.379	0.00
**Reproductive system**						
Self-compatible	0.6547	7.483	0.00	0.5781	6.383	0.00
Self-incompatible/ Dioceous	0.4645	5.114	0.00	0.1799	2.714	0.01
**Dispersal mode**						
Abiotic	0.3238	3.858	0.00	0.4851	5.322	0.00
Biotic	-0.01092	0.829	0.41	0.00575	1.081	0.29
